# Disparities in diabetes mellitus among Caribbean populations: a scoping review

**DOI:** 10.1186/s12939-015-0149-z

**Published:** 2015-02-25

**Authors:** Nadia R Bennett, Damian K Francis, Trevor S Ferguson, Anselm JM Hennis, Rainford J Wilks, Eon Nigel Harris, Marlene MY MacLeish, Louis W Sullivan, On behalf of the U.S. Caribbean Alliance for Health Disparities Research Group (USCAHDR)

**Affiliations:** Epidemiology Research Unit, Tropical Medicine Research Institute, The University of the West Indies, Kingston, West Indies Jamaica; Chronic Disease Research Centre, Tropical Medicine Research Institute, The University of the West Indies, Bridgetown, West Indies Barbados; The University of the West Indies, Kingston, West Indies Jamaica; Department of Medical Education, Morehouse School of Medicine, Atlanta, USA; The Sullivan Alliance, Alexandria, USA

**Keywords:** Diabetes, Health disparities, Caribbean, Afro-Caribbean, Blacks

## Abstract

**Background:**

Despite the large body of research on racial/ethnic disparities in health, there are limited data on health disparities in Caribbean origin populations. This review aims to analyze and synthesize published literature on the disparities in diabetes mellitus (DM) and its complications among Afro-Caribbean populations.

**Methods:**

A detailed protocol, including a comprehensive search strategy, was developed and used to identify potentially relevant studies. Identified studies were then screened for eligibility using pre-specified inclusion and exclusion criteria. An extraction form was developed to chart data and collate study characteristics including methods and main findings. Charted information was tagged by disparity indicators and thematic analysis performed. Disparity indicators evaluated include ethnicity, sex, age, socioeconomic status, disability and geographic location. Gaps in the literature were identified and extrapolated into a gap map.

**Results:**

A total of 1009 diabetes related articles/manuscripts, published between 1972 and 2013, were identified and screened. Forty-three studies met inclusion criteria for detailed analysis. Most studies were conducted in the United Kingdom, Trinidad and Tobago and Jamaica, and used a cross-sectional study design. Overall, studies reported a higher prevalence of DM among Caribbean Blacks compared to West African Blacks and Caucasians but lower when compared to South Asian origin groups. Morbidity from diabetes-related complications was highest in persons with low socioeconomic status. Gap analysis showed limited research data reporting diabetes incidence by sex and socioeconomic status. No published literature was found on disability status or sexual orientation as it relates to diabetes burden or complications. Prevalence and morbidity were the most frequently reported outcomes.

**Conclusion:**

Literature on diabetes health disparities in Caribbean origin populations is limited. Future research should address these knowledge gaps and develop approaches to reduce them.

## Introduction

In 1995, the global prevalence of diabetes mellitus (DM) in adults was estimated to be 4.0% and projected to rise to 5.4% by the year 2025 [1]. However by 2011, the International Diabetes Federation (IDF) estimated the global prevalence of diabetes mellitus to be 8.3% and projected a rise to 9.9% by 2030. In absolute numbers, this translates to 366 million persons with diabetes mellitus in 2011 which will rise to 552 million people by 2030. Eighty percent of those with diabetes live in low and middle income countries [2]. In the Caribbean, the overall prevalence of diabetes mellitus is estimated to be approximately 9% [3] and is responsible for 13.8% of all deaths among adults in the region [2]. Diabetes mellitus is therefore one of the major public health challenges for the Caribbean in the twenty-first century.

Researchers have found that patterns in allocation of resources and differential access to care directly influence health in population sub-groups [4]. This has led to the emergence of the study of these differences or health disparities as a major focus of research and public health policy over the last two decades. These changes in focus are reflected in policies such as the in the United States Healthy People 2010/2020, aimed at eliminating health disparities [5,6] as well as the World Health Organization World Conference on Social Determinants of Health in 2011 resulting in a political declaration and commitment for the implementation of the social determinants of health approach to reduce health disparities [7].

The literature defines health disparities as *“the variation or differences in health status resulting from the distribution of the effects of health determinants between and among different population groups”* [8]. In addition health disparities imply a social disadvantage among population-subgroups as it relates to a particular health outcome such, as morbidity, mortality or access to care. These health disparities can occur by gender race or ethnicity, education or income, disability, living in rural localities or sexual orientation (disparity indicators) [9].Through measurement of the indicators of health, the degree of disparity can be characterized by absolute and relative differences in measures of occurrence captured as proportions, rates and ratios (disparity measures).

The Caribbean is a geographically diverse region and its citizens live both inside and outside of the region. It includes islands in the Caribbean Sea, but for the purpose of this paper was expanded to include some South and Central American countries (Guyana, Suriname, and Belize) and islands in the Atlantic (Turks and Caicos Islands) which through strong historical, political, and social links and are part of the Caribbean Community (CARICOM). The Caribbean population is predominantly of African descent, but includes an admixture of peoples representing South Asians, Chinese, Europeans, and people from the Middle East. The racial admixture varies between countries; for example, in Jamaica and Barbados over 90% of the population is of African descent while in Trinidad and Tobago and Guyana over 50% of the population are of South Asian origin or mixed ethnicity.

There is a paucity of information on disparities in diabetes mellitus within populations of Afro-Caribbean ethnicity. Scoping reviews have emerged as a method which “aims to rapidly map the key concepts underpinning a research area and the main sources and types of evidence available. It can be undertaken as stand-alone projects, especially where an area is complex or has not been reviewed comprehensively before” [10]. The scoping review is one method of knowledge synthesis which differs from other types of literature reviews in that it addresses broader topics, while a systematic review focuses on specific questions on a relatively narrow range of quality assessed studies. Like the systematic review but unlike other traditional literature reviews, the scoping review employs a systematic replicable approach which includes a search strategy to reduce bias.

This scoping review aimed to summarize the published studies on disparities in diabetes mellitus in Afro-Caribbean populations in order to identify gaps in the available literature as well as characterize the factors which might explain the disparities observed.

The specific objectives were:

1. To review and synthesize the published evidence on health disparities in diabetes mellitus among Afro-Caribbean origin populations
2. To evaluate the effect of health disparities on outcomes including incidence and prevalence of diabetes type1 and 2, micro-vascular or macro-vascular complications of diabetes, and mortality related to diabetes mellitus
3. To identify which health disparity indicators are more frequently reported among Caribbean populations and identify gaps in the literature on health disparities in diabetes mellitus.

## Methods

A scoping review was undertaken in accordance with the framework published by Arksey and O’Malley [11].

### Inclusion criteria

Studies that reported on diabetes mellitus and the effect which health disparities had on Caribbean populations were examined. Disparity indicators included were: age, sex, ethnicity/race, geographic location, sexual orientation, disability status and socioeconomic status. Disability status was defined as a physical or mental permanent inability to carry out routine function, and socioeconomic status was measured by occupation, education, income, or household amenities. Study participants had to be adults 18 years or older, of Caribbean origin, living in CARICOM or Caribbean immigrant populations living outside of the Caribbean. The complete list of included countries is shown in Appendix 1. Outcomes assessed included incidence and prevalence of diabetes, micro-vascular or macro-vascular complications of diabetes, mortality related to diabetes mellitus and utilization and access to health services among persons with diabetes.

### Exclusion criteria

We excluded studies which did not report on an Afro-Caribbean population or immigrant populations of Caribbean descent alone or as a comparator group with other populations (e.g. African American, UK-Africans). Studies reporting only on diabetes control (e.g. blood glucose levels or glycosylated haemoglobin) and studies which grouped Afro-Caribbean populations with other ethnic groups e.g. West African or Latin American so that separate effects could not be determined were also excluded. We also excluded studies in which the less than 18 year old age group could not be separated from those older than 18 years of age.

### Types of outcome measures

Both absolute and relative differences in measures of occurrence estimated as proportions rates and ratios were extracted as well as any qualitative information found.

### Search strategy

A comprehensive search strategy was developed in consultation with a library and information science specialist. The search was designed to retrieve all articles combining the concepts of ‘Caribbean region’ , ‘African ancestry’ and ‘black Caribbean ethnicity’ with specific chronic diseases, and social determinants of health, health disparities, or health inequity in relevant bibliographic databases. The following databases were searched:

- Ovid MEDLINE(R) <1946 to June 20, 2013>)
- Ovid MEDLINE(R) In-Process & Other Non-Indexed Citations <1946 to June 20, 2013>)
- CENTRAL via Cochrane Library (February 2013)
- LILACS
- PsycINFO 1806 to June 2013.

For conference proceedings, theses, dissertations and other grey literature the following databases were searched:

- Science Citation Index Expanded (SCI-EXPANDED) --1992-present
- Social Sciences Citation Index (SSCI) --1992-present
- Arts & Humanities Citation Index (A&HCI) --1992-present
- Conference Proceedings Citation Index- Science (CPCI-S) --1992-present
- Conference Proceedings Citation Index- Social Science & Humanities (CPCI-SSH) --1992-present
- Proquest: Theses and Dissertation 1990-present.

The search was conducted without a study design filter in order to retrieve qualitative as well as quantitative papers. The search was limited to the English language.

### Screening and charting

Duplicated articles were identified and removed from the database prior to screening. The titles and abstracts of articles identified by the search strategy were independently screened for relevance by two review authors according to the inclusion and exclusion criteria described above. Citations were managed using EndNote X5 and Microsoft Excel. Discrepancies between review authors were resolved through discussion and, where necessary, by consultation with a third review author. Studies meeting the criteria outlined were charted using a standard study extraction form with domains as listed in Appendix 2. Textual data were charted using Microsoft Excel. The charting approach was akin to a ‘narrative review’ [12] to include detailed information of population characteristics according to identified indicators of health disparities. Reported study quality and limitations of each included study were also charted.

### Data synthesis

The synthesis of the charted data was conducted on two levels as suggested by Arskey and O’Malley [11]. Firstly, a numerical analysis was conducted to determine the extent, nature and distribution of the studies included in the review. The included studies were used to create tables and charts, mapping the distribution of studies according to geographic location; study design; publication year; outcome measures used to quantify disease occurrence; and disparity indicators and outcome. This process subsequently informed the approach to identifying main areas of research and the development of gap maps. Secondly, textual data charted in excel were organized thematically, according to the disparity indicators. The review findings were then organized into categories which combined diabetes related outcomes and disparity indicators.

## Results

**Figure 1:**
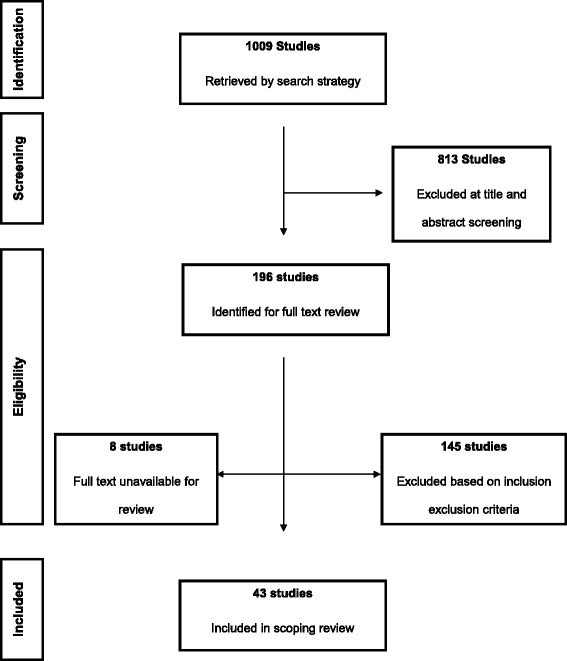
**Summary of the inclusion and exclusion process.**

**Figure 2:**
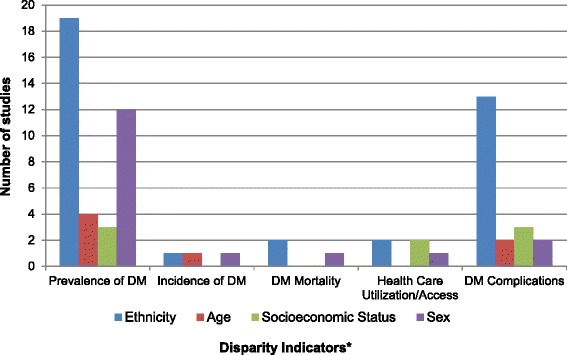
**Number of included studies according to disparity measures and types of diabetes outcome addressed.**

One thousand and nine (1009) published studies on diabetes and health disparities were identified from the search. After title and abstract screening of these references 196 studies met the criteria for full text review, from which 43 studies were selected for final analysis. Details of the screening and study selection process are shown in Figure 1. Table 1 shows the characteristics of the included studies including study design, country of origin, setting, and quality and limitations of included studies. Figure 2 summarizes the distribution of included studies with regards to the disparity measures addressed and the type of diabetes related outcome.

**Table 1:** **Characteristics of studies included in analysis**

**Author/Year**	**Study Design**	**Study Characteristics**	**Ethnic group/Location**	**Country/Region**	**Setting**	**Reported Quality/Limitations**
Abbott, 2005 [13]	Cross-sectional study	15,646	Afro-Caribbean vs. Asians vs. Whites	United Kingdom	Community health center	No limitations reported. Direct standardization use to calculate age adjusted rates.
		Men: 8574				
		Women: 6892				
		Type 1&2 DM				
Abbott, 2011 [14]	Cross-sectional study	15,692	Afro-Caribbean vs. South Asians vs. Whites	United Kingdom	Community based population study	No limitations reported.
		Male: 8448				
		Female: 7236				
		Mean age: 61 ± 14.0y				
		Type 1 & 2 DM				
Admiraal, 2011 [15]	Cross-sectional study	1,443	Hindustani Surinamese vs. African Surinamese vs. Dutch Caucasians	Netherlands	Community based	Discrepancy in measurement of physical activity as a confounder between ethnic groups. No adjustment for other known confounders (diet).
		Age: 35-60y				
		Type 2 DM				
Agyemang, 2011 [16]	Cross-sectional study	3,386	South East Asian Indians vs. Afro-Caribbean in England and Netherlands	United Kingdom, Netherlands	Population based	Lack of data on all the important explanatory variables that might contribute to the observed differences, such as diet, psychosocial stress, and early-life exposures. Furthermore, there was a lack of valid data on other types of physical activity and socioeconomic position measures.
		Male: 1474				
		Female: 1912				
		Type 2 DM				
Babwah, 2006 [17]	Cross-sectional study	360	Trinidad and Tobago	Trinidad and Tobago	Urban clinic	Reporting bias, lack of multivariate analysis to adjust for known confounders (SES)
		Male: 93				
		Female: 267				
		Age >13 years				
		Type 2 DM				
Barcelo, 2006 [18]	Cross-sectional study	10,587	Barbados vs. Mexico	Caribbean, Latin America	Population-based	No limitation stated. Prevalence adjusted for known confounders.
		Male: 4041				
		Female: 6546				
		DM unspecified				
Baskar, 2006 [19]	Cross-sectional study	6,047	Afro-Caribbean vs. Caucasian vs. Indo-Asian	United Kingdom	Community based	No limitation stated. Analysis adjusted for known confounders
		Male: 3359				
		Female 2688				
		Type 1 & 2 DM				
Cappuccio, 1997 [20]	Cross-sectional study	1,578	Afro-Caribbean vs. West African vs. UK Whites vs. Asians	United kingdom	Community based, general practice	Selection bias in Caribbean group and low response rate. Prevalence rates age standardized by direct method.
		Age: 40-59				
		Type 2 DM				
Chaturvedi, 1996 [21]	Cohort study	227	Afro-Caribbean vs. European (UK)	United Kingdom	Hospital based	Small sample size particularly among African Caribbeans. Inability to conduct sex specific analysis. No collection of important confounders. Prevalence rates were age standardized.
		Male : 122				
		Female: 105				
		Age: 35-55				
		Type 2 DM				
Conway, 2003 [22]	Cross-sectional study	832	Afro Caribbean vs. Whites vs. Indo-Asian	United Kingdom	Hospital based study	Adjustment for known confounders carried out.
		Male: 449				
		Female: 383				
		Age: 74 ± 12y				
		DM unspecified				
Cooper, 1997 [23]	Cross-sectional study	4,823	African origin populations in Nigeria, St. Lucia, Barbados, Jamaica, the United States, and the United Kingdom	Barbados, Jamaica, Nigeria, St Lucia, United Kingdom, United States of America	Community based	Limited sample size in some sites.
		Age: 25-74y				
		Type 2 DM				
Cox, 2011 [24]	Cross-sectional study	87	Jamaica	Jamaica	Hospital based	No limitations stated.
		Male: 35				
		Female: 52				
		Age 40-90y				
		DM				
Creatore, 2012 [25]	Cohort Study	3,927,059	Immigrant populations in Canada	Canada	Population based	Due to data restrictions analyses were not adjusted for risk factors. Immigration data restricted sample to immigrants to Canada between 1985 and 2000.
		Male: 2,094,042				
		Female: 1,833,017				
		Age : >40 yrs.				
Cruickshank, 1980 [26]	Cross-sectional study	27,667	Jamaican vs. White vs. West Indian Black	United Kingdom	Hospital based	No limitations stated.
		Male: 11,157				
		Female: 9,235				
		Age 30-59y				
		DM unspecified				
Cruickshank, 1987 [27]	Case–control study	282	Afro-Caribbean vs. Caucasian vs. Asian	United Kingdom, Jamaica	Hospital based clinic attendees	No limitations stated. No clear description of statistical technique.
		Men: 119				
		Women: 163				
		DM unspecified				
Eldemire, 1996 [28]	Cross-sectional study	1,318	Jamaica	Jamaica	Population based	No limitations stated and no clear description of statistical techniques.
		Male: 649				
		Female: 669				
		Age >60				
		Type 2 DM				
Ferguson, 2011 [29]	Cross-sectional study	2,848	Jamaica	Jamaica	Community based	No limitations stated. Appropriate adjustment for confounders.
		Age: 15-74				
		DM unspecified				
Florey, 1972 [30]	Cross-sectional study	696	Jamaica	Jamaica	Community based	No limitation stated and no clear description of data analysis technique.
		Male: 329				
		Female: 367				
		Age 25-64y				
		DM unspecified				
Gill, 2011 [31]	Cross-sectional study	5,354	Afro-Caribbean vs. South Asians	United Kingdom	Clinic based screening programme	Low response rate (49.6%). Age sex adjustments were not conducted due to small number of cases.
		Male: 2544				
		Female: 2810				
		Age > 45 y				
		DM unspecified				
Goyal, 2007 [32]	Cohort study	271	Afro Caribbean vs. Whites vs. South Asians	United Kingdom	Community clinic setting	No limitations stated.
		Male: 184				
		Female: 87				
		Age				
		Type unspecified				
Gulliford, 1997 [33]	Cross-sectional study	1,149	Afro-Trinidadian vs. Indo-Trinidadian	Trinidad and Tobago	Hospital based	Evidence of selection bias with more ill patients less likely to provide interview data.
		Male: 454				
		Female: 695				
		Age >15y				
		DM unspecified				
Gulliford, 1998 [34]	Cross-sectional study	622	Afro-Trinidadian vs. Indo-Trinidadian	Trinidad and Tobago	Health center	Sample biased to socially less advantage individuals.
		Male: 204				
		Female: 418				
		DM type 2				
Gulliford, 2001 [35]	Cross-sectional study	2,117	Afro-Trinidadian vs. Indo-Trinidadian	Trinidad and Tobago	Government health centres	Large geographically representative sample. Reporting bias; over-reporting of private utilization in older age group.
		Male: 633				
		Female: 1484				
		DM unspecified				
Gulliford, 2004 [36]	Cross-sectional study	548	Indo Trinidadian vs. Afro Trinidadian vs. mixed Trinidadian	Trinidad and Tobago	Population based community study	Higher non-response among affluent groups. Appreciable risk of type II error in findings among men.
		Male: 250				
		Female: 298				
		Age >25				
		DM type 2				
Gulliford, 2010 [37]	Cross-sectional study	31,484	Afro-Caribbean vs. Whites vs. Africans vs. Other blacks	United Kingdom	Clinic based screening programme	Missing data. Analysis adjusted for multiple factors.
		Male: 16,145				
		Female: 15,339				
		DM type 1 & 2				
Khattar, 2000 [38]	Cohort study	688	Afro-Caribbean vs. South Asians vs. Whites	United Kingdom	Hospital and community based	Retrospective design with some degree of information bias from missing data. No mention of statistical procedures for missing data.
		Male 436				
		Female: 249				
		DM unspecified				
Leggetter, 2002 [39]	Case–control study	528	Afro-Caribbean vs. European	United Kingdom	Hospital based	Limitations to the quality of data collected retrospectively.
		Age >30				
		DM type 1 & 2				
Leske,1999 [40]	Cross-sectional study	4,631	Black vs. White vs. Mixed	Barbados	Community based population	No stated limitations. Limited description of fata analysis.
		Male ; 1991				
		Female: 2640				
		Age: 40-84y				
		DM unspecified				
Markus, 2007 [41]	Cohort study	1,200	African vs. Afro-Caribbean	United Kingdom	Hospital based	Case Ascertainment bias in study population. Adjustment for known confounders such as socioeconomic status reported.
		Male: 671				
		Female: 529				
		DM unspecified				
Mbanya, 1999 [42]	Cross-sectional study	1,481	African vs. Afro-Caribbean	Jamaica, United Kingdom, Cameroon	Community based	Relatively small sample available for British African-Caribbeans. Overall response rate of 66%. Age standardization of data for comparison across populations.
		Male: 706				
		Female: 775				
		Age: 27-74y				
		DM unspecified				
Miller, 1996 [43]	Cohort study	2,491	Trinidad and Tobago	Trinidad and Tobago	Population based	No limitations stated. Sex specific incidence rates calculated with adjustment for age and ethnic group alone and then with additional adjustment for other factors.
		Male: 1386				
		Female:1105				
		Age 35-69y				
		DM type 2				
Molokhia, 2011 [44]	Cohort study	832	Trinidad and Tobago	Trinidad and Tobago	Population based	Authors reported cohort study design as the only limitation due to single village cohort. Analyses were adjusted for known risk factors and survival analysis adjusted for age and sex.
		Male 349				
		Female: 483				
		Age >20				
		DM unspecified				
Mungrue, 2011 [45]	Cohort study	81	Trinidad and Tobago	Trinidad and Tobago	Hospital based	Major limitation was poor record keeping and therefore the unavailability of all the data which also in part contributed to restricting the study to only one site. No survival analysis reported due to small sample size. Relevant confounders were collected and included in analysis.
		Male: 44				
		Female: 37				
		Age 10-79				
Prasad, 2004 [46]	Cohort study	465	Afro-Caribbean vs. South Asians vs. Whites	United Kingdom	Clinic or hospital based study	No limitation stated. Statistical methods vaguely described.
		Male: 288				
		Female: 177				
		DM unspecified				
Riste, 2001 [47]	Cross-sectional study	1,022	Afro-Caribbean vs. Whites vs. Pakistani	United Kingdom	Population based register	Statistical methods included standardization for cross comparisons and log transformation carried out for variables with clearly skewed distribution. No limitations were reported.
		Male:502				
		Female: 520				
		Age 25-79				
		DM type 2				
Sedgwick, 2003 [48]	Cross-sectional study	1,899	Afro-Caribbean vs. Whites vs. Black African	United Kingdom	Clinic or hospital based study	Subjects were preferentially selected from GP practices in areas with a high proportion of ethnic minorities in order to increase the representation of these groups. There was some evidence of differential non-response by ethnic minority subjects.
		Male: 409				
		Female: 390				
		Age				
		DM type 2				
Shantsila, 2011 [49]	Cross-sectional study	128	Afro-Caribbean vs. South Asians vs. Whites	United Kingdom	Not stated	One limitation of the study is the relatively few Afro-Caribbean subjects. There were difficulties in Afro-Caribbean subjects who met inclusion criteria, and many of them were reluctant to participate in this research. Analyses were adjusted for clinical and demographic variables.
		Male :110				
		Feamle:18				
		Age				
		DM unspecified				
Sharp, 2008 [50]	Randomized controlled study	509	Afro-Caribbean vs. Whites	United Kingdom	clinical trial	No limitations stated.
		Male: 441				
		Female: 68				
		Age: 40-79				
		DM type 2				
Sosin, 2008 [51]	Cross-sectional study	108	Afro-Caribbean vs. South Asians vs. Whites	United Kingdom	Clinic or hospital based study	Recruitment of African Caribbean subjects fell short of the numbers required from our power calculation. Limitation of cross sectional study design.
		Male: 85				
		Female: 23				
		DM unspecified				
Sargeant, 2002 [52]	Cohort Study	728	Jamaica	Jamaica	Population based	Lack of data for two important confounders, physical activity and diet.
		Male: 290				
		Female: 438				
		Age: 25-74				
		DM type 2				
UKPDS-32, 1998 [53]	Cohort study	4,974	Mixed	United Kingdom	Clinic or hospital based study	No stated limitations. Analyses adjusted for known confounders.
		Men: 2920				
		Women: 2054				
		Age 25-65y				
Wilks, 1999 [54]	Cross-sectional study	1,303	Jamaica	Jamaica	Population based	No limitations stated.
		Male: 520				
		Female: 783				
		Age:25-74y				
		DM type 2				
Wilks, 1998 [55]	Other	9772	Nigeria vs. Caribbean vs. United Kingdom vs. United States of America	Barbados, Jamaica, Nigeria, St Lucia, United Kingdom, United States of America	Population survey	No limitations stated.
		Male:4581				
		Female: 5191				
		Age >25				
		DM type 2

### Prevalence and incidence of diabetes in Afro-Caribbean populations

Seventeen papers reported on the prevalence of diabetes mellitus by ethnicity [15,16,18,20,22,23,25,31,32,38,40-42,47,49-51] Overall, the prevalence of diabetes was higher among Afro-Caribbean populations when compared to other African ethnic groups [25,41,42], except in one study where the prevalence among Black Africans (11%) was higher in comparison to Afro-Caribbean (7%) (Table 2) [23]. Markus et al. reported a significantly higher prevalence of diabetes in Afro-Caribbean (45.1%) vs. African (30.8%) ethnic groups among patients with a history of stroke [41]. The prevalence of diabetes in the Afro-Caribbean groups were notably higher in comparison to Caucasians as reported by 13 studies [15,20,22,23,25,32,38,40,42,47,49-51]. However, only 6 of these studies showed any statistically significantly difference (Table 1). The baseline populations in which these prevalence estimates were reported varied widely from the general population to those with heart failure or hypertension. When Afro-Caribbean groups were compared to Asians, the results were mixed, [31] Gill showed the prevalence of diabetes was higher in Afro-Caribbeans (31% vs. 26%; p < 0.05) and in contrast Goyal [32] reported a higher prevalence in South Asians (27.5% vs. 15.6%; p < 0.05).

**Table 2:** **summary of findings for differences in outcome measures by ethnic group**

**Author & Year**	**Study characteristics**	**Ethnicity**			
		**African Caribbean**	**Caucasian**	**Black Africans**	**Asian/Hispanic**
***Prevalence (%)***					
Admiraal, 2011 [15]	General population	12.4^‡^	6.7	-	-
Ageymang, 2011 [16]		PR: Male 1.97;	1	-	-
		Female 1.90			
Barcelo, 2006 [18]	Elderly	21.6	-		21.5
Cappuccio, 1997 [20]	General population	17.9^‡^	6.7		25.4
Conway, 2003 [22]	Atrial fibrillation and stroke	42^‡^	15.0	-	41.0
Cooper, 1997* [23]	General population	7.2	UK: 10.8	10.6	-
			US: 10.6		
Creatore, 2012* [25]	General population	9.5	5.1	7.9	13.0
Gill, 2011 [31]	Minority population	31^†^	-	-	26.0
Goyal, 2007 [32]	Suspected coronary artery disease	15.6^†^	12.0	-	27.5
Khattar, 2000 [38]	Essential hypertension	15.0^‡^	5.0	-	17.0
Leske, 1999* [40]	General population	19.4	7.5	-	-
Markus, 2007 [41]	Stroke population	45.1^√^	-	30.8	-
Mbanya, 1999 [42]	General population	10.6	14.0	2.8	-
Riste, 2001 [47]	General population	Male: 23.4	Male: 20.8		Male: 29.9
		Female: 20.8	Female: 19.9		Female: 35.7
Shantsila, 2011 [49]	Systolic heart failure	64.0^‡^	30.0	-	62.0
Sharp, 2008 [50]	Hypertension	38.0^‡^	19.0	-	
Sosin, 2008* [51]	Systolic heart failure	41.0	23.0	-	44.0
***Incidence (rates)***					
Miller, 1996 [43]	General population	Male: 12.5^†^	-	-	Male: 23.6
		Female: 14.4			Female: 22.7
***Mortality (HR)***					
Chaturvedi, 1996 [21]	Persons with type 2 diabetes	0.42 (0.24, 0.76)^‡^	1.0	-	-

PR-prevalence ratio; HR – Hazard ratio.

-No comparison.

^†^p < 0.05 Afro-Caribbean vs. South Asian.

^‡^p < 0.05 Afro-Caribbean vs. Caucasian.

^√^p < 0.05 Afro-Caribbean vs. Black Africans.

*significance not reported/interpretable.

Only one study reported on incident diabetes by ethnicity [43] The authors found that there was a higher incidence of diabetes in Indo-Trinidadian men compared to Afro-Trinidadian men but not for women.

### Age and Sex differences in diabetes

Four studies reported on the prevalence of diabetes by age groups. Overall, the prevalence of diabetes increased with age across all ethnic groups and social indicators [18,28,36,54]. In age groups < 35 the prevalence was ~2% and in those >65 years it ranged between 10 and 16%.

Eleven papers investigated the sex differences in the prevalence of diabetes [18,23,25,28-30,34,36,42,44,54], of which 6 noted a higher prevalence among women compared to men which ranged between 9.3 - 14% vs. 6.4-9.8% respectively [23,28-30,36,54]. One study evaluated the burden of diabetes among immigrants in Canada and reported higher prevalence of newly diagnosed diabetes among men (10.0%) compared to women (9.3%) [25]. This difference was not statistically significant. Four studies noted no sex differences in diabetes prevalence [18,34,42,44].

Only one study was found which reported sex differences in incident diabetes and found that sex was not a significant predictor of incident diabetes [52].

### Socioeconomic inequalities and diabetes

Few studies addressed socioeconomic status and diabetes. Among the studies reviewed we found that the prevalence of diabetes was higher among persons with lower incomes and lower educational attainment. This trend in the prevalence of diabetes was similar across the Caribbean [18,36].

**Figure 3:**
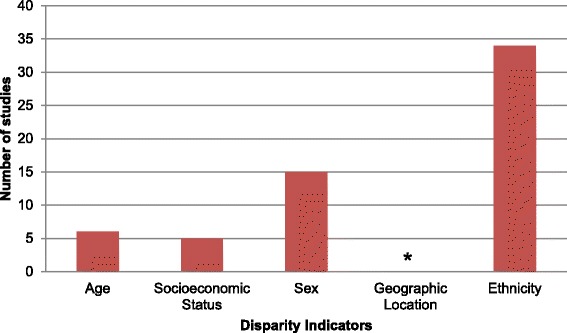
**Frequency of included studies by disparity measure.** * no study was reported on geographic location.

Our search found no studies addressing the prevalence of diabetes using other indicators of disparity, such as, geographical location, rural vs. urban distribution or by disability status among the Afro-Caribbean ethnic group (see Figure 3).

### Diabetes mortality in Afro-Caribbean populations

Three studies reported on mortality attributable to diabetes of which one noted ethnic differences between Afro-Caribbeans and UK Caucasians [21,44,53]. Chaturvedi investigated the differences in morbidity and mortality due to non-insulin dependent diabetes in Afro-Caribbeans and Europeans and found that Afro-Caribbean’s had lower (HR 0.42 (0.24, 0.76, p < 0.05) all-cause mortality compared with the Europeans [21]. Molokhia reported that there was a higher rate of mortality attributable to diabetes in women (22.1%) when compared to men (5.8%) [44].

### Disparities in diabetes complications

Twelve studies investigated disparities in micro-vascular and macro-vascular complications among persons with diabetes [13,14,19,24,27,33,34,37,39,40,45,46,48]. The micro-vascular complications included retinopathy, nephropathy as well as peripheral sensory neuropathy or was not individually specified in the papers reviewed. Studies reporting on macro-vascular complications specified diabetic foot amputations only.

Afro-Caribbeans had a higher prevalence of micro-vascular complications related to diabetes when compared to South Asians and Caucasians in the United Kingdom [19,27,37]. In one study it results were mixed depending on the method of testing for the peripheral sensory neuropathy [13] and in another study that compared Afro-Caribbeans to Caucasians the neuropathy was lower (23 ± 4 vs. 35 ± 3 *p = 0.03)* though nephropathy (14 ± 3 vs. 11 ± 2 *p = 0.6*) and retinopathy (24 ± 4 vs. 20 ± 3 *p = 0.4*) were higher respectively [39]. Overall macro-vascular complications related to diabetes was lower in the Afro-Caribbean populations compared to Caucasians [13,19,39] but comparable to the South-Asian ethnic group [13,19]. Within the Caribbean, the rates of amputation however were found to be higher in Afro-Trinidadians compared to Indo-Trinidadians [34].

With regard to age and sex differences in diabetes complications, one hospital-based cross sectional study among persons with amputation in Jamaica found that older males had higher rates of below knee amputation [24]. In the same study, women were found to have significantly better quality of life and function scores than men as measured by the SF-36 [24]. In another study Leske et al. reported that although the prevalence of diabetic retinopathy varied by age, an increase with age was only evident in women [40].

### Inequalities in healthcare utilization and access and diabetes complications

There were very little published data in this area. One study found that there were significant differences in the burden of diabetes complications in the Caribbean population with higher levels of morbidity and lower healthcare utilization in those of lower socioeconomic status [33]. Morbidity from diabetes was greater in groups with lower educational attainment. Private health care was used less frequently by persons in the lower social groups. In another paper [37], Gulliford’s group found that in comparing the Afro-Caribbean, Caucasian, African and other Black ethnic groups, there was notable socioeconomic inequality in sight threatening diabetic retinopathy.

### Place of residence: urban rural differences

There remains a dearth of published information on the influence of place of residence on diabetes mellitus (see Figure 3). Of the studies found, most were carried out in urban areas and among those which included rural dwellers; the authors did not present data on differences in disease outcome by place of residence.

### Disparity indicators and knowledge gaps

**Figure 4:**
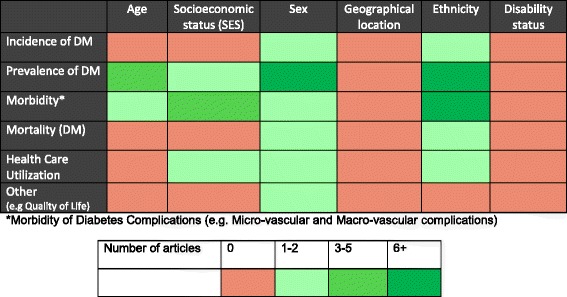
**Health disparities research gaps identified in diabetes literature.**

The most frequently reported disparity indicators among the afro-Caribbean population in this review were ethnicity and sex (Figure 3). Among studies reporting on ethnic disparities, the majority examined the prevalence of diabetes (19) and its complications (13). With the exception of incidence of diabetes, sex differences were explored by 15 studies across the other disparity measures, the bulk of which were prevalence studies. Very few studies reported on socioeconomic status and age by the disparity measure, while no study was found to examine indicators such as geographic location and disability status (Figure 4). Of note, very few indicators were investigated in terms of incidence, and mortality from diabetes.

## Discussion

This review presents an in-depth outline of the scope of the published literature relating to the investigation of disparities in diabetes mellitus in the Caribbean and the Caribbean Diaspora. The review was based on a comprehensive search of the literature and as such should capture the full range of available studies on health disparities in the Caribbean. We acknowledge however that it is possible that some relevant studies may have been missed from this review, as the search strategy, although iterative and broad, was restricted only to studies published in English which may exclude literature published in the Spanish and French speaking Caribbean as well as those from Caribbean immigrant populations published in languages other than English.

Most of the studies were done in urban settings on Afro-Caribbean immigrants to the United Kingdom over a forty year period from 1972–2012. The comparison groups were mainly Afro-Caribbean vs. Caucasians and Afro-Caribbean vs. South Asians. Very few studies published in English were found which compared Afro-Caribbeans to Latin American populations and none of the studies compared Afro-Caribbean to African American populations. The lack of comparison with the African American population was mainly due to the fact that the categories of race provided by the U.S. Office of Management and Budget and used by the Census Bureau do not disaggregate the Black/African American to allow for comparisons using subset groups such as African-Caribbean population, and would account for no studies comparing the Afro-American population with the Afro-Caribbean Population [56].

Overall it can be said that, the prevalence of diabetes mellitus was higher within the Afro-Caribbean population when compared to Caucasian or other African populations but lower when compared to South Asian populations which were the main groups of comparison. The prevalence of diabetes is higher in women when compared to men and higher in people of lower SES when measured by education and or income. In addition, the morbidity and mortality from diabetes was higher in lower SES groups which also reflected accessibility of health care. The prevalence of the micro-vascular complications related to diabetes mellitus is higher in people of Afro-Caribbean descent when compared to other ethnic groups but when the macro-vascular complications were considered the results were mixed across the ethnic groups.

Although disparities in diabetes mellitus as it relates to disability were of interest, no studies on this area were found.

It must be noted that there was a wide range of study types looking at different populations of persons with diabetes so many of the comparisons were difficult and inferences had to be made.

## Conclusions

We have found that while a number of studies have been published exploring health disparities in relation to diabetes mellitus, the literature on diabetes health disparities in Caribbean origin populations is limited, in particular as it relates to studies conducted within the Caribbean. There were no studies comparing Afro-Caribbean with African American populations. Such studies would help in understanding the mechanisms underlying health disparities among minority population in the United States and the influence of factors such as self-governance, discriminations and variations in health care systems on health disparities. Future research should address these knowledge gaps and approaches to reduce them as we seek to reduce health disparities and improve health for all social and ethnic groups.
